# Lung Ultrasound in Rheumatoid Arthritis–Associated Interstitial Lung Disease: Association with Radiological Patterns, Lung Function Tests, and Disease Activity—A Single-Center Retrospective Cross-Sectional Study

**DOI:** 10.3390/biomedicines14102281

**Published:** 2026-10-08

**Authors:** Tiziana La Blasca, Giorgio Monteleone, Rosangela Di Liberti, Giacomo Barbata, Vittorio Lo Turco, Lidia La Barbera, Giuliana Guggino, Riccardo Messina, Nicola Scichilone

**Affiliations:** 1 Division of Respiratory Medicine, Department PROMISE “Paolo Giaccone” University Hospital, 90143 Palermo, Italy; tizianalb40@gmail.com (T.L.B.); giorgio.monteleone@unipa.it (G.M.); rosangeladiliberti@gmail.com (R.D.L.); giacomobarbata7@gmail.com (G.B.); vittorioloturco@gmail.com (V.L.T.); riccardomessina82@gmail.com (R.M.); 2 Center for Interstitial and Rare Lung Diseases, Department of Pneumology, Ruhrlandklinik, University Hospital Essen, University of Duisburg-Essen, 45239 Essen, Germany; 3 Rheumatology Section, Department PROMISE, “Paolo Giaccone” University Hospital, 90143 Palermo, Italy; lidialb90@gmail.com (L.L.B.); giuliana.guggino@unipa.it (G.G.)

**Keywords:** rheumatoid arthritis, interstitial lung disease, lung ultrasound, LUS score, usual interstitial pneumonia pattern, disease activity score 28-C reactive protein

## Abstract

**Introduction:** Interstitial lung disease (ILD) represents one of the most relevant extra-articular manifestations in rheumatoid arthritis (RA), and significantly contributes to morbidity and mortality. High-resolution computed tomography (HRCT) is the gold standard for the diagnosis of ILD; however, its use in daily practice is limited. Lung ultrasound (LUS) is a non-invasive, repeatable, and bedside tool for ILD assessment. This study aimed to evaluate the association between quantitative LUS score and HRCT findings in RA patients. **Methods:** We conducted a single-center, retrospective cross-sectional study in RA patients with and without ILD. Demographic, clinical, lung function, and disease activity (DAS28-CRP) data were collected, together with LUS and HRCT findings at baseline. Correlation and regression analyses were performed to evaluate associations between LUS score, HRCT patterns, pulmonary function, and disease activity. **Results:** Thirty-seven RA subjects (M/F: 20/17; mean age 68.8 ± 10.4 years) were included; ILD was present in 24 patients (64.8%), including 17 (45.9%) with a UIP pattern. LUS score inversely correlated with DLCO (r = −0.413; *p* = 0.01) and positively with age (r = 0.47; *p* = 0.003) and DAS28-CRP (r = 0.366; *p* = 0.02). Higher LUS score were significantly associated with UIP pattern at both uni- (OR 1.09; 95%CI 1.02–1.16; *p* < 0.01) and multivariable regression analysis adjusted for age (OR 1.07; 95%CI 1.01–1.15; *p* = 0.02). Likewise, DAS28-CRP was significantly associated with a UIP pattern on chest HRCT in both univariable (OR 3.05, 95% CI 1.30–7.14; *p* = 0.01) and multivariable analyses adjusted for age (OR 2.35, 95% CI 1.19–6.86; *p* = 0.02). **Conclusions:** Overall, higher LUS score values were associated with reduced pulmonary function, higher DAS28-CRP, and the presence of a UIP pattern on chest HRCT. However, these findings should be considered exploratory and require further confirmation in larger prospective studies.

## 1. Introduction

Rheumatoid arthritis (RA) is a chronic systemic autoimmune disease characterized by persistent synovial inflammation and progressive joint destruction, frequently accompanied by extra-articular manifestations that substantially contribute to morbidity and mortality [1,2]. Among the pulmonary manifestations associated with RA—including pleuritis, pulmonary nodules, bronchiectasis, and small airway disease—rheumatoid arthritis–associated interstitial lung disease (RA-ILD) represents the condition with the greatest impact on survival and long-term prognosis [3,4]. RA-ILD is a heterogeneous clinical entity resulting from the complex interplay between immune-mediated inflammatory mechanisms and chronic fibrotic remodeling processes [5]. The disease may present either as clinically overt ILD or as a subclinical condition, with parenchymal abnormalities detectable even in the absence of significant respiratory symptoms [6,7,8]. On chest high-resolution computed tomography (HRCT), the most commonly observed radiological patterns are usual interstitial pneumonia (UIP) and non-specific interstitial pneumonia (NSIP). In particular, the UIP pattern has been associated with greater functional impairment, a more rapid decline in pulmonary function, and poorer survival outcomes compared with predominantly inflammatory patterns [9,10]. Within the diagnostic work-up of RA-ILD, chest HRCT remains the gold standard for the detection and characterization of interstitial lung involvement, enabling accurate assessment of the distribution, extent, and severity of parenchymal abnormalities while also providing relevant prognostic information for clinical management [11,12]. However, the routine use of HRCT is limited by radiation exposure, costs, and reduced feasibility for repeated evaluations, particularly in patients with early or subclinical disease. Among pulmonary function tests, the diffusing capacity of the lung for carbon monoxide (DLCO) plays a pivotal role in the early detection of interstitial involvement and in monitoring disease progression. Reduced DLCO represents one of the most sensitive indicators of early interstitial damage, and has been shown to correlate with both the extent of parenchymal abnormalities and clinical outcomes in patients with RA-ILD [7,13]. Given the limitations of HRCT, lung ultrasound (LUS) has recently emerged as a promising, non-invasive bedside imaging technique, free of ionizing radiation and potentially useful in the evaluation of ILD [14]. LUS is based on the interpretation of acoustic artifacts generated at the pleural–lung interface, reflecting alterations in the air-to-tissue ratio within subpleural regions. In the presence of interstitial abnormalities, increased tissue density and reduced alveolar air content leads to the appearance of vertical reverberation artifacts (B-lines), pleural line irregularities, and small subpleural consolidations, which are considered indirect signs of interstitial involvement [15,16,17]. Several studies have demonstrated the high sensitivity of LUS in detecting ILD-related abnormalities, with significant correlations reported between LUS findings, HRCT features, and pulmonary function test (PFTs) [14]. Nevertheless, the role of LUS in RA-ILD is still limited and complementary to HRCT, and its relationship with HRCT patterns, pulmonary function impairment, and systemic disease activity has not yet been fully established [18,19]. Furthermore, systemic inflammatory is a possible driver of lung involvement in RA [9]. Particularly, higher disease activity has been associated with increased risk of ILD, and persistent inflammation may contribute to fibrotic remodeling leading to the development of UIP pattern [9,10]. To date, whether disease activity measured by disease activity score28-C reactive protein (DAS28-CRP) is associated with the HRCT pattern has not been clarified. In this context, the present study aimed to evaluate the association between LUS score, HRCT findings, pulmonary function, and disease activity in a highly selected patients with RA who underwent HRCT due to clinical suspicion of ILD. Additionally, we investigated the association between DAS28-CRP and the UIP pattern on chest HRCT.

## 2. Materials and Methods

### 2.1. Study Design

A single-center retrospective cross-sectional observational study was conducted at the Respiratory Medicine Unit of the University Hospital “Paolo Giaccone” in Palermo, Italy. Consecutive adult patients (≥18 years) with a diagnosis of RA according to the 2010 ACR/EULAR classification criteria, who attended the outpatient clinic and underwent high-resolution computed tomography (HRCT) because of respiratory symptoms, abnormal PFTs, or clinical suspicion of lung involvement, between 2021 and 2026, were retrospectively identified from our institutional database and considered for inclusion [20]. This resulted in a highly selected, ILD-enriched cohort in which the prevalence of ILD was expected to be higher than in the general RA population. Clinical characteristics potentially associated with an increased risk of rheumatoid arthritis–associated interstitial lung disease (RA-ILD), including older age, RA-related autoantibody positivity, and higher articular activity assessed by DAS28-CRP were also recorded and considered as part of the clinical assessment. At the time of HRCT, the presence of RA-ILD was assessed. All patients with suspected or established RA-ILD were discussed within a multidisciplinary team (MDT) as a part of diagnostic and clinical assessment. Clinical, functional, and HRCT findings were integrated during the multidisciplinary discussion to assess the presence of ILD and characterize its radiological pattern. This was not a systematic HRCT screening study of an unselected RA cohort; rather, patients were identified from routine clinical practice according to the clinical indications leading to HRCT evaluation.

### 2.2. Eligibility Criteria

Patients were eligible whether they met all the following criteria: age ≥ 18 years; RA diagnosed according to the 2010 ACR/EULAR classification criteria; LUS performed within the study protocol; complete PFTs (spirometry and diffusing lung capacity for carbon monoxide, DLCO) performed within 2 months of LUS; chest HRCT available within 6 months of LUS; and DAS28-CRP obtained during rheumatological evaluation, performed within 6 months from the pneumological assessment. Rheumatological and pneumological assessments took place during separate visits. LUS was performed using a SonoScape P9 Lite ultrasound system (SonoScape Medical Corp., Shenzhen, China). PFTs were performed using a Vyntus™ BODY plethysmograph (Jaeger Medical, Höchberg, Germany).

Patients were excluded if they had: ILD unrelated to RA, acute respiratory infection at the time of clinical or LUS assessment, significant chronic pulmonary disease unrelated to RA (e.g., severe chronic obstructive pulmonary disease or severe asthma), or incomplete clinical, functional, or radiological data, including examinations performed outside the predefined study time windows. Patients with a documented diagnosis of another connective tissue diseases (including Sjögren’s disease) were excluded. Concomitant rheumatic diseases were not systematically screened for; hence, undiagnosed overlap conditions cannot be ruled out. Moreover, ILD was classified as RA-associated when the MDT, integrating clinical, serological, functional, and HRCT data considered RA as the most likely cause and found no evidence of alternative etiology (i.e., other connective tissue diseases, drug toxicity, exposure). Cases attributed by the MDT to a condition unrelated to RA were classified as non-RA-ILD and excluded. 

### 2.3. Data Collection

Baseline demographic data (i.e., age, sex, and body mass index, BMI), as well as clinical characteristics, including comorbidities, PFTs results, and HRCT patterns, were collected at first presentation. Serological variables, including anti-cyclic citrullinated peptide antibody and rheumatoid factor levels, were also recorded at baseline. At the same visit, LUS was calculated, while RA and disease activity was assessed using the DAS28-CRP.

### 2.4. HRCT Assessment

HRCT images were jointly reviewed by two radiologists with expertise in ILD and classified by consensus during multidisciplinary discussion (MDD), according to the predefined classification criteria, and considering clinical, radiological, and functional information. The UIP and probable UIP patterns were defined according to the ATS/ERS/JRS/ALAT guidelines, whereas the other radiological patterns (i.e., non-specific interstitial pneumonia, NSIP, organizing pneumonia, OP, and unclassifiable ILD, uILD) were identified by following the ERS/ATS criteria [21,22]. Because a consensus diagnosis was reached through joint review rather than independent assessment by each radiologist, formal interobserver agreement was not assessed. Additionally, radiologists were blinded to LUS findings, thereby minimizing the risk that HRCT evaluation was influenced by ultrasound results. On the other hand, they were not blinded to disease activity and PFTs, which were part of the clinical assessment during the MDD. This lack of blinding to clinical and functional data as well as the absence of interobserver agreement for HRCT and intra- or interobserver reproducibility for LUS are acknowledged as potential sources of bias.

### 2.5. Lung Ultrasound Assessment

All patients underwent LUS performed by an experienced single operator using a high-end ultrasound system equipped with a 2–5 MHz convex transducer and a 7–12 MHz linear transducer. Because only one LUS examination per patient was available and no repeated examinations were performed, formal intra- or interobserver reproducibility analyses could not be performed. The convex transducer was used for the systematic assessment of all 14 lung zones, whereas the high-frequency linear transducer was used, when required, for a more detailed evaluation of the pleural line and superficial subpleural lesions. Examinations were performed with the patient in either the sitting or supine position according to a standardized 14-zone scanning protocol, including the anterior, lateral, and posterior regions of both hemithoraces.

Each hemithorax was systematically examined in a cranio-caudal direction along the parasternal, midclavicular, anterior axillary, posterior axillary, midscapular, and paravertebral lines. Ultrasound settings were optimized for lung imaging using an abdominal preset adapted for thoracic examination. Acoustic power was reduced by 50%, while gain, focal zone, and imaging depth were adjusted to optimize visualization of the pleural line and vertical artifacts. Images were acquired in the longitudinal intercostal plane with the transducer positioned perpendicular to the intercostal spaces. For each lung zone, the pleural line appearance, the presence and distribution of B-lines, and the presence of pleural irregularities or subpleural consolidations were recorded. When subpleural consolidations were detected, patients were instructed to perform deep inspiratory maneuvers to assess the presence of a dynamic air bronchogram.

**Figure 1:**
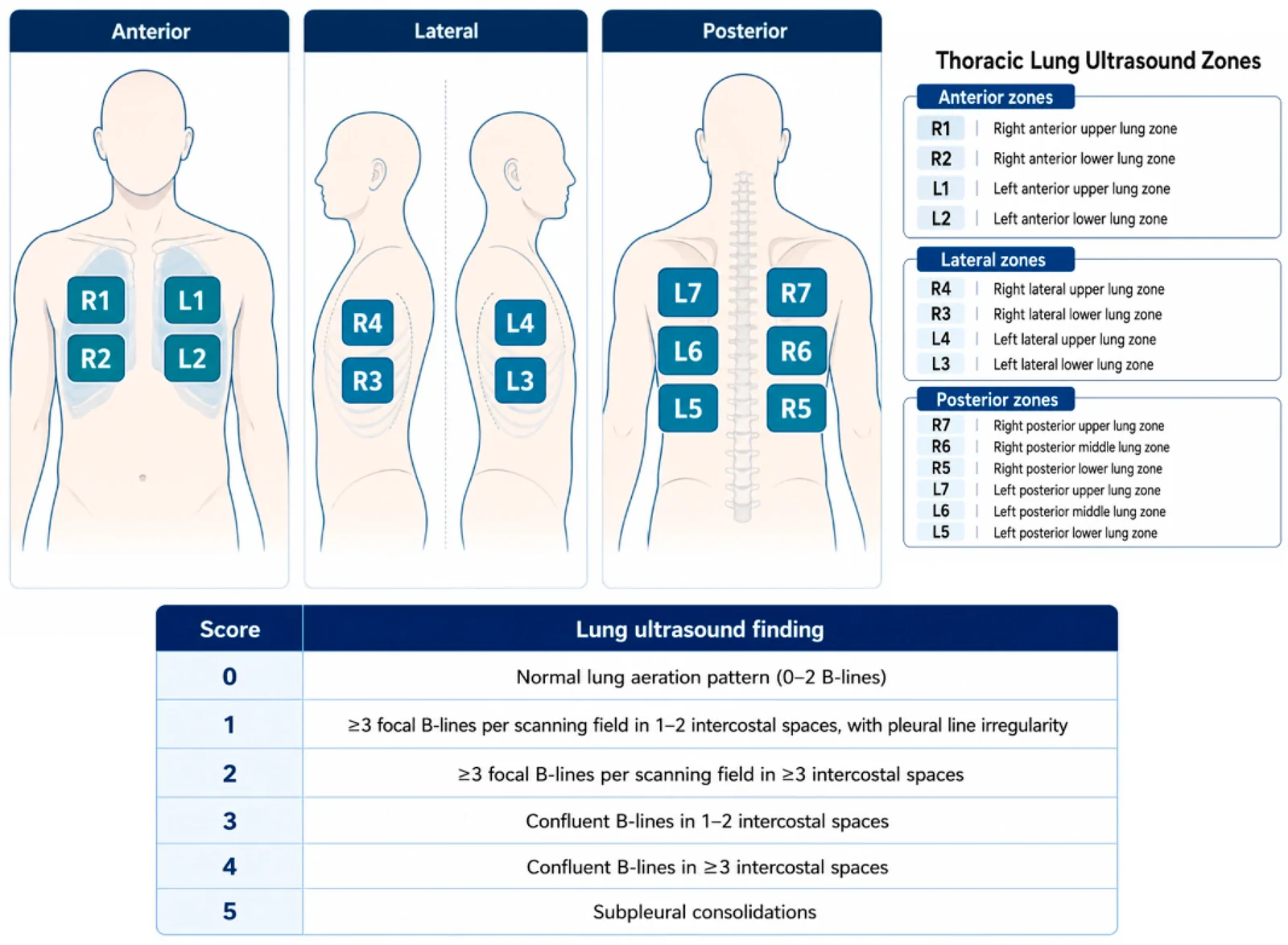
Lung ultrasound examination protocol and scoring system. Lung ultrasound was performed using a 14-zone scanning protocol covering the anterior, lateral, and posterior regions of both hemithoraces with a 2–5 MHz convex transducer. A 7–12 MHz linear transducer was used when required for detailed assessment of the pleural line and superficial subpleural lesions. Each lung zone was examined in the longitudinal intercostal plane, evaluating pleural line morphology, B-lines, pleural irregularities, and subpleural consolidations. LUS findings were scored for each zone as follows: 0 = normal aeration (0–2 B-lines); 1 = ≥3 focal B-lines in one or two intercostal spaces; 2 = ≥3 focal B-lines in three or more intercostal spaces; 3 = confluent B-lines in one or two intercostal spaces; 4 = confluent B-lines in three or more intercostal spaces; and 5 = subpleural consolidation. The total LUS score was obtained by summing the scores from all 14 lung zones (range 0–70), providing a semiquantitative measure of the extent of pulmonary ultrasound abnormalities.

This 14-zone scanning protocol was adapted from previously validated LUS assessment methods described in connective tissue disease–associated ILD [23,24]. LUS findings were graded using a semiquantitative scoring system applied to each of the 14 lung zones: 0, normal aeration (0–2 B-lines); 1, ≥3 focal B-lines per scan in one or two intercostal spaces; 2, ≥3 focal B-lines per scan in three or more intercostal spaces; 3, confluent B-lines in one or two intercostal spaces; 4, confluent B-lines in three or more intercostal spaces; and 5, subpleural consolidation. The global LUS score was calculated as the sum of the scores from all 14 lung zones, yielding a total score ranging from 0 to 70. This score was used as a semiquantitative measure of the extent of pulmonary ultrasound abnormalities (Figure 1).

### 2.6. Study Outcomes

First, we examined the relationships between quantitative LUS score and HRCT patterns in highly selected RA patients undergoing HRCT because of clinical suspicion of ILD. Second, we investigated the relationship between LUS score, PFTs, and disease activity assessed by DAS28-CRP. Third, as a secondary, exploratory analysis, we evaluated the association between DAS28-CRP and the UIP pattern on chest HRCT.

### 2.7. Statistical Analysis

The normality of data distribution was assessed using the Kolmogorov–Smirnov test. Continuous variables were expressed as mean ± standard deviation or median interquartile range (IQR), where IQR range indicates 75th minus 25th percentile according to their distribution. Differently, categorical variables were presented as frequencies and percentages. Correlations between LUS score, demographic characteristics, lung function test parameters, and DAS28-CRP were evaluated using Pearson correlation coefficient. As the primary analysis, uni- and multivariable logistic regression was used to assess the association between LUS score and DAS28-CRP, as continuous variables, and the presence of a UIP pattern on chest HRCT, adjusting for age. As a secondary, exploratory analysis, LUS score and DAS28-CRP were dichotomized according to their cohort median values, and their association with HRCT patterns was assessed using the chi-square test. A *p*-value < 0.05 was considered statistically significant. Statistical analyses were performed using Jamovi (The Jamovi Project, 2025; version 2.7.2).

## 3. Results

**Figure 2:**
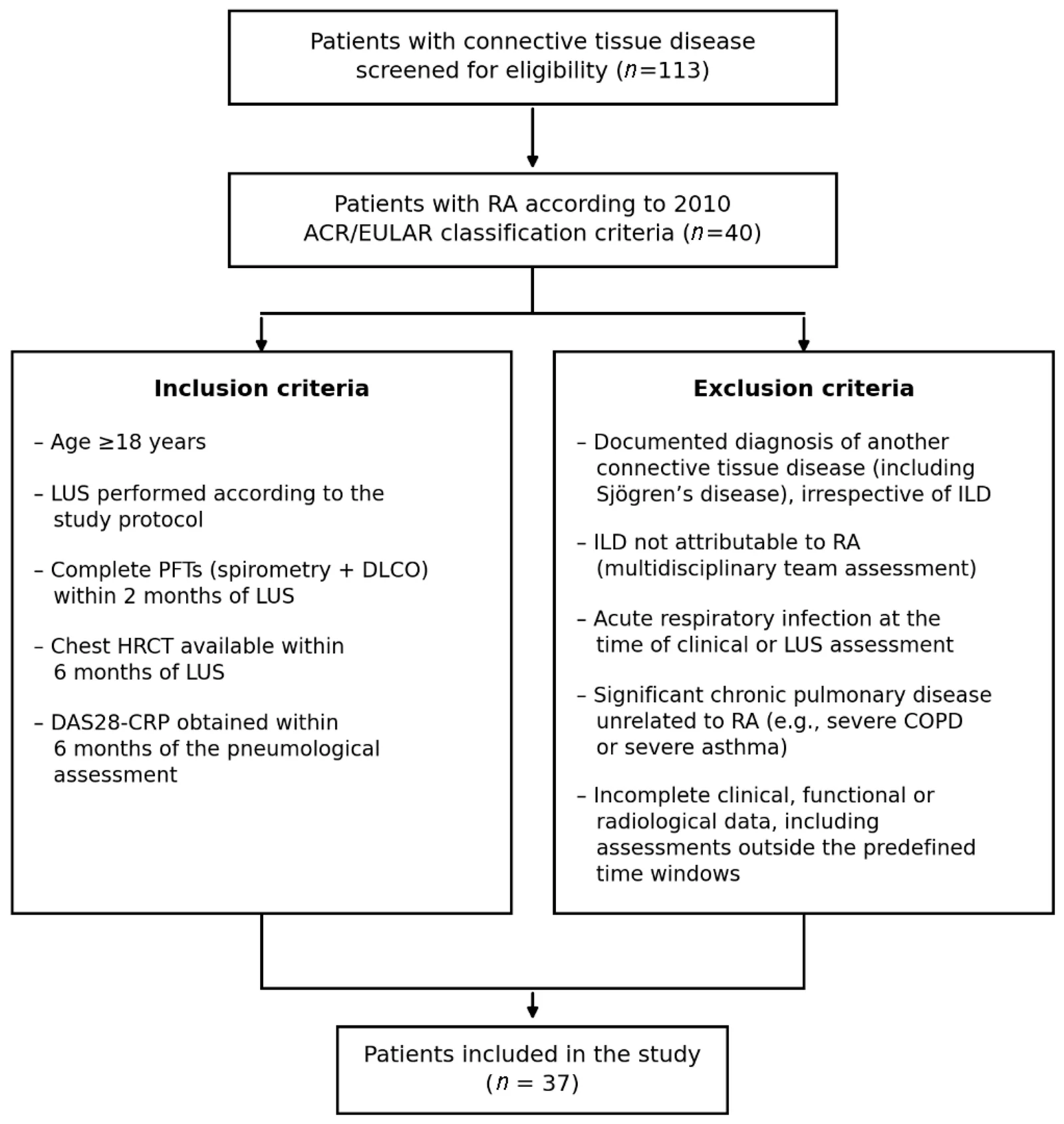
Study flowchart. Of 113 patients with connective tissue disease screened for eligibility, 40 patients fulfilled the 2010 ACR/EULAR classification criteria for RA. Thirty-seven patients met all eligibility criteria and were included in the final analysis.

Among 113 patients diagnosed with connective tissue disease, 40 patients had RA diagnosis. Of them, 37 RA patients (M/F 20/17, mean age: 68.8 ± 10.4 years) met eligibility criteria and were included in the final analysis (Figure 2). Baseline characteristics of study population are summarized in Table 1. Additionally, distributions of LUS score across HRCT subgroups are reported in Supplementary Material S1.

### 3.1. Baseline Correlation Between LUS Score, DAS28-CRP, Demographics, and Lung Function Tests

**Figure 3:**
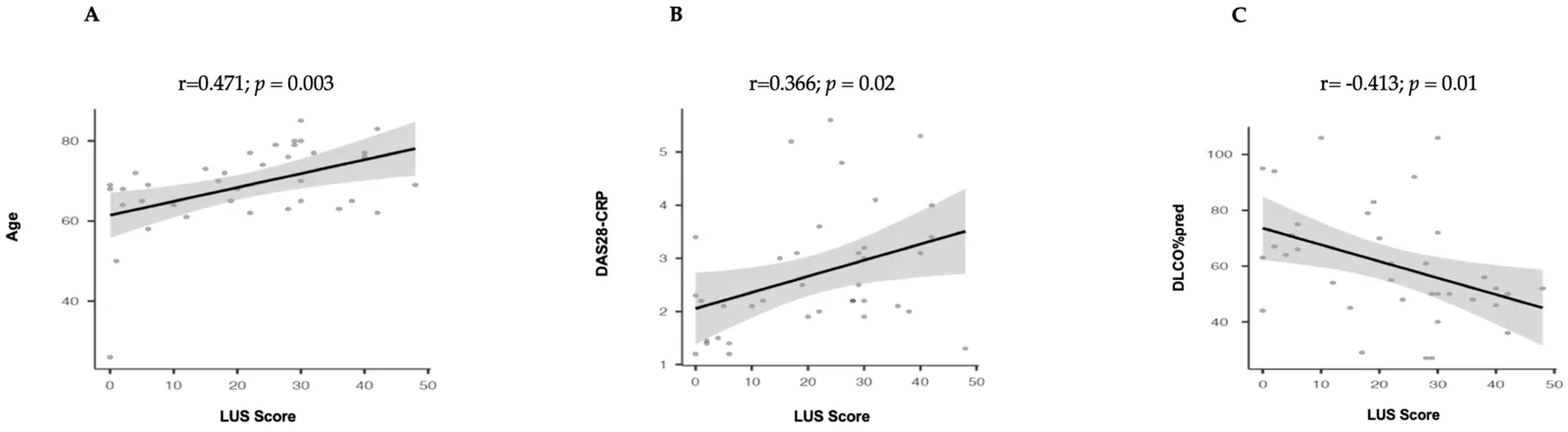
(**A**–**C**). Correlation between LUS score, disease activity, and pulmonary function parameters. (**A**) LUS score was positively correlated with age (r = 0.47; *p* = 0.003). (**B**) LUS score had a positive correlation with DAS28-CRP (r = 0.366; *p* = 0.02). (**C**) LUS score had negative correlation with DLCO % predicted (r = −0.413; *p* = 0.01).

At baseline, a significant correlation between LUS score and age (r = 0.471; *p* = 0.003) and DAS28-CRP (r = 0.366; *p* = 0.02) was found (Figure 3A,B). In contrast, no statistically significant correlation was observed between LUS score and BMI (*p* = 0.17). LUS score demonstrated an inverse correlation with DLCO % predicted (r = −0.413; *p* = 0.01), whereas no significant correlations were found between LUS score and FVC, either in absolute values (*p* = 0.31) or as percentage (*p* = 0.43) of predicted (Figure 3C). DAS28-CRP had no correlation with age (*p* = 0.18) and BMI (*p* = 0.85). Likewise, DAS28-CRP was not correlated with DLCO% pred (*p* = 0.10) and FVC both absolute (*p* = 0.52) and %pred (*p* = 0.33).

### 3.2. Association Between LUS Score and DAS28-CRP with Radiological Patterns

**Figure 4:**
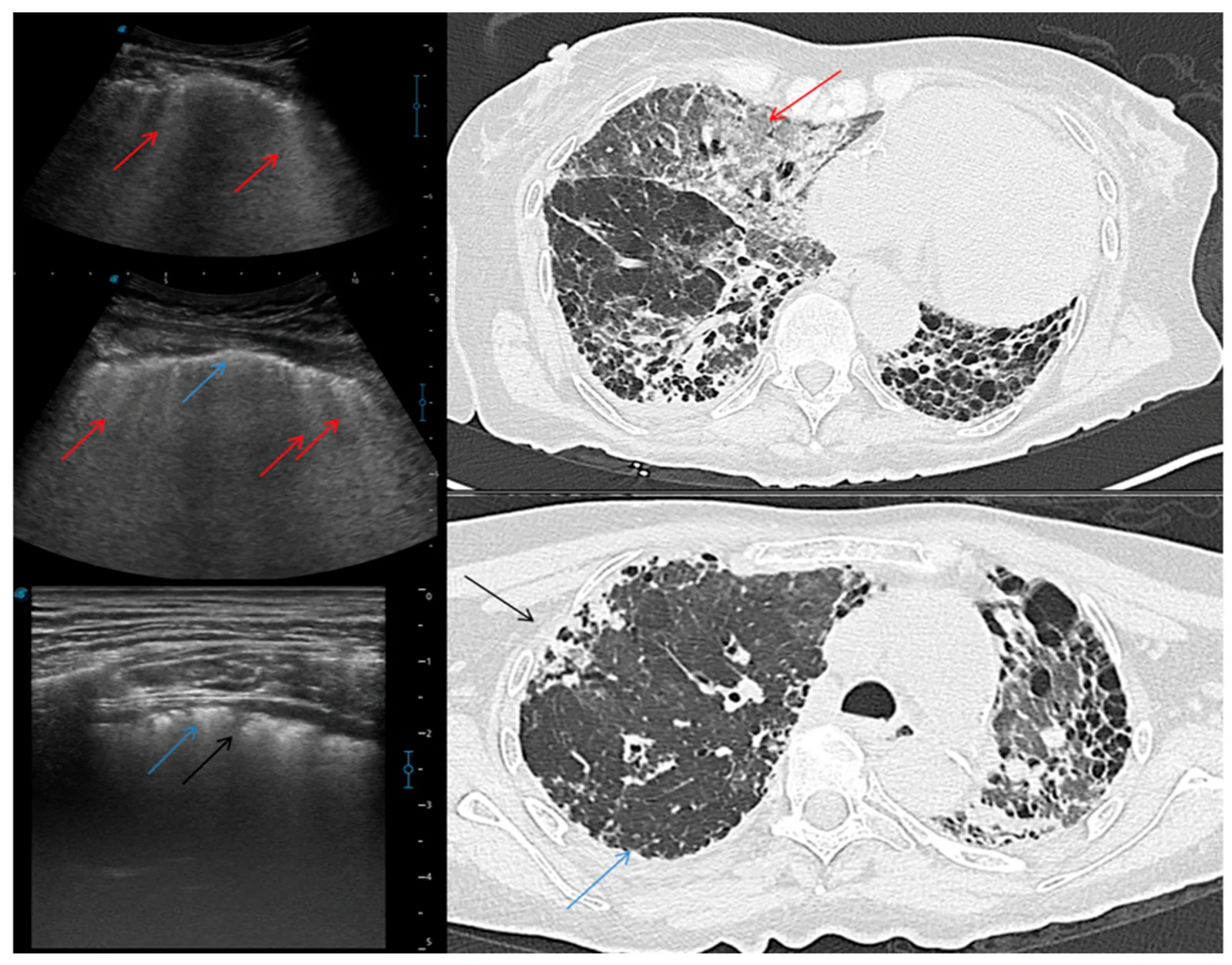
Correspondence between lung ultrasound (LUS) and high-resolution computed tomography (HRCT) findings in patients with rheumatoid arthritis–associated interstitial lung disease (RA-ILD) and a UIP pattern on chest HRCT. The images demonstrate corresponding LUS and HRCT findings, including B-lines (red arrows), pleural line irregularities (blue arrows), and subpleural consolidations (black arrows). These sonographic abnormalities reflect characteristic peripheral lung changes that can be detected by LUS in interstitial lung involvement.

At univariable logistic regression analysis, a LUS score was associated with UIP pattern on chest HRCT (OR 1.09; 95%CI: 1.02–1.16; *p* < 0.01). These association remained significant at multivariable analysis after adjustment for age (OR 1.07; 95%CI: 1.01–1.15; *p* = 0.02) (Table 2). Similarly, DAS28-CRP was significantly associated with UIP pattern on chest HRCT at univariable analysis (OR 3.05; 95%CI: 1.30–7.14; *p* = 0.01), retaining its independent association after adjustment for age at multivariable analysis (OR 2.35; 95%CI: 1.19–6.86; *p* = 0.02) (Table 3). The comparison between LUS and HRCT findings regarding UIP pattern is shown in Figure 4.

### 3.3. Exploratory Analyses Using Median-Based Cut-Offs to Assess Association Between LUS Score, DAS28-CRP, and UIP Pattern

Then, we performed an exploratory analysis using median-based, non-validated cut-offs to further investigate the association between radiological patterns and LUS score and DAS28-CRP; the latter were dichotomized according to their median values (22 and 2.20, respectively). The analysis demonstrated a statistically significant association between the UIP radiological pattern and higher categorized LUS scores (χ^2^ = 10.1; *p* < 0.01), as well as between the UIP pattern and higher categorized DAS28-CRP values (χ^2^ = 5.45; *p* = 0.02) (Supplementary Material S2).

In contrast, no significant associations were observed between the NSIP pattern and either LUS score (*p* = 0.77) or DAS28-CRP (*p* = 0.37). Likewise, the uILD pattern on chest HRCT was not significantly associated with LUS score (*p* = 0.35) or DAS28-CRP (*p* = 0.42); the organizing pneumonia pattern showed no significant association with either LUS score (*p* = 0.35) or DAS28-CRP values (*p* = 0.19). Similarly, a LUS score ≥ 22 was significantly associated with a UIP pattern, both at uni- (OR 10.89, 95% CI 2.26–52.4, *p* < 0.01) and multivariable analysis adjusted for age (OR 9.31, 95% CI 1.64–52.8, *p* = 0.01) (Supplementary Material S3; Table S1A). Similarly, DAS28-CRP ≥2.20 was associated with the UIP pattern at univariable (OR 5.70, 95% CI 1.24–26.2, *p* = 0.02) and multivariable analysis (OR 4.96, 95% CI 1.03–23.8, *p* = 0.04) (Supplementary Material S3; Table S1B). However, the wide confidence intervals observed for these estimates indicate substantial imprecision, likely due to the small sample size and should be interpreted with caution. Similarly, the absence of association between other radiological patterns (i.e., NSIP, OP, and uILD) may be explained by the very small sample size of these subgroups. 

## 4. Discussion

Over the last few years, LUS has become an increasingly valuable bedside, radiation-free modality for the dynamic assessment of pulmonary and pleural conditions [25]. Beyond its established diagnostic and procedural applications, its role is progressively being explored in the evaluation and monitoring of interstitial lung involvement. However, within the ILD landscape, current guidelines do not support its replacement of HRCT for ILD screening or characterization. In particular, the latest European Respiratory Society/European Alliance of Associations for Rheumatology (ERS/EULAR) clinical practice guidelines for connective tissue disease–associated ILD do not recommend substituting HRCT with LUS for ILD screening in patients with RA, reflecting the current limitations of ultrasound in disease characterization and pattern definition [26]. Similarly, the American College of Rheumatology/CHEST recommendations endorse PFTs and HRCT for the assessment of patients with systemic autoimmune rheumatic diseases at risk of ILD, emphasizing the integration of functional, radiological, and clinical data during follow-up [27]. In this context, LUS may represent a complementary, accessible, and reproducible tool to support clinical decision-making and to identify patients who may benefit from further diagnostic evaluation. In our study, we investigated the relationship between semiquantitative LUS assessment, chest HRCT findings, pulmonary function parameters, and systemic disease activity in highly selected patients with RA undergoing HRCT because of clinical suspicion of ILD.

First, we found an inverse correlation between total LUS score and DLCO % predicted, whereas no significant correlation was observed with FVC. While FVC reflects global lung volume restriction, DLCO is often affected earlier by impairment of gas exchange, including interstitial thickening, loss of alveolar–capillary surface area, pulmonary vascular involvement, emphysematous changes, or combinations of these mechanisms [28]. Accordingly, DLCO may be a more sensitive endpoint in early RA-ILD phenotype and may decline before overt restrictive ventilatory defects become evident; in RA-ILD, reduced DLCO has been associated with greater disease extent, functional deterioration, and adverse outcomes [6,7,12]. These findings suggest that LUS-detected abnormalities may parallel impairment in gas exchange as reflected by DLCO. This relationship may be explained by the ability of LUS to detect indirect signs of peripheral air–tissue interface alteration, including B-lines, pleural abnormalities, and subpleural consolidations, which reflect increased subpleural tissue density and interstitial changes affecting the alveolar–capillary interface [15,16,17,25]. However, DLCO is also influenced by emphysema, pulmonary vascular disease, anemia, and smoking and, therefore, both reduced DLCO and LUS abnormalities cannot be interpreted as markers of interstitial fibrosis. 

On the other hand, we observed a positive correlation between total LUS score and RA disease activity assessed by DAS28-CRP. This finding is consistent with the concept of RA-ILD as part of a systemic autoimmune condition characterized by chronic inflammation and progressive tissue remodeling [9,10]. Higher DAS28-CRP values may therefore reflect a more severe systemic phenotype in which articular and pulmonary involvement coexist. DAS28-CRP is a composite measure incorporating articular disease activity, patient-reported global assessment, and CRP which is designed to assess joint disease activity [29]. Nevertheless, it does not specifically capture pulmonary involvement, this association should not be interpreted as evidence of a direct causal relationship between articular activity and ILD. Therefore, these findings should be interpreted with caution, as the individual components of the DAS28-CRP were not systematically available in our dataset, and we could not determine which component primarily contributed to the observed association. In our cohort, higher LUS score was associated with an increased likelihood of exhibiting a UIP pattern, even after adjustment for age. This association could be driven by the shared dependence of both LUS and UIP on subpleural disease distribution, as UIP in RA-ILD predominantly affects peripheral lung regions that are directly assessed by LUS through B-line generation. These findings align with those reported by Vermant et al., who demonstrated in a prospective cohort of RA patients that LUS B-lines were more strongly associated with HRCT-defined interstitial abnormalities than clinical symptoms or FVC, supporting the role of LUS in detecting subclinical pulmonary involvement [30]. Similarly, Ottaviani et al. reported good performance of LUS as a screening tool for RA-ILD, showing that the presence of more than five B-lines yielded good diagnostic accuracy and was significantly associated with both HRCT findings and PFTs [31]. Importantly, LUS does not allow direct visualization of key HRCT features of UIP, such as honeycombing, nor does it reliably distinguish UIP from other interstitial patterns or quantify disease extent with the accuracy of HRCT and, subsequently, cannot replace multidisciplinary radiological assessment. Likewise, B-lines are non-specific artifacts that can reflect not only in inflammatory or fibrotic interstitial changes but also in hydrostatic pulmonary edema or other causes of increased extravascular lung water. Conversely, the lack of statistically significant associations with NSIP, uILD, or OP may be due to the small sample size of these subgroups and should be further investigated in adequately powered studies.

A further relevant observation was the association between RA disease activity, as measured by DAS28-CRP, and the presence of a UIP pattern on chest HRCT. Higher DAS28-CRP values were associated with an increased risk of UIP pattern even after adjustment for age. This finding is in line with previous studies demonstrating that greater RA disease activity may be associated with an increased risk of developing RA-ILD and with worse outcomes among affected patients. In particular, Sparks et al. reported a progressive increase in RA-ILD risk across increasing levels of disease activity, while Assayag et al. identified disease activity as a predictor of mortality in patients with RA-ILD [32,33]. To our knowledge, however, no previous study has specifically examined the relationship between DAS28-CRP and the UIP pattern. Nevertheless, the cross-sectional design of the study as well as the lack of adjustment for several important determinants such as disease duration, smoking exposure, and treatment represent relevant limitations that render this association hypothesis-generating and requires further confirmation in larger, prospective studies.

Overall, these results suggest that greater LUS abnormalities may be associated with a more severe pulmonary phenotype, despite LUS ≥22 and DAS28-CRP ≥2.20 being median-derived cut-off that should be regarded as cohort-derived, exploratory, and non-validated thresholds, particularly given their wide confidence intervals, which indicate substantial statistical instability. Hence, these analyses should be considered secondary and should not be interpreted as supporting clinical thresholds or decision-making rules. Although LUS cannot replace HRCT for disease characterization or pattern recognition, its association with reduced DLCO, higher disease activity, and the UIP pattern might support its potential role as a complementary and readily available bedside tool for the clinical assessment in highly selected RA patients with increased suspicion of ILD. However, the cross-sectional nature of the present study and the limited sample size, particularly within individual HRCT pattern subgroups, preclude conclusions regarding causality. Therefore, larger prospective studies are warranted to determine whether LUS findings and DAS28-CRP may be associated with the presence and severity of UIP pattern at chest HRCT, and to clarify their potential integration into multimodal screening and follow-up strategies. 

Our study presents several limitations. First, the retrospective, single-center, cross-sectional design and the relatively small sample size limit the robustness and generalizability of our findings. Second, the high prevalence of ILD and UIP in our cohort likely reflect an ILD-enriched population rather than an unselected RA population, potentially introducing selection and spectrum bias, and further limiting extrapolation to the broader RA population. This encompassed a small number of patients within non-UIP subgroups (i.e., NSIP, OP, uILD), which severely limits statistical power and renders subgroup-specific findings strictly exploratory. Then, although predefined intervals were applied, LUS, PFTs, HRCT, and DAS28-CRP were not assessed simultaneously, introducing variability related to changes in disease status over time. Moreover, despite the use of a standardized protocol, LUS remains an operator-dependent technique, and the lack of complete blinding to clinical information together with the absence of formal reproducibility represent an additional source of potential observer bias. Additionally, threshold values used (LUS score ≥22 and DAS28-CRP ≥2.20) were non-validated and derived from the cohort median, and used for exploratory purposes only. Given the limited number of UIP cases and consequent uncertainty in the estimates, these findings should be considered hypothesis-generating and future ROC curve analyses in larger cohorts will be necessary to establish clinically validated cut-offs. Furthermore, chest X-ray was not systematically performed or consistently available, precluding direct comparison with LUS. Finally, the absence of longitudinal follow-up prevented evaluation of the prognostic value of LUS and its ability to predict ILD progression. Larger, prospective, multicenter studies with standardized and simultaneous assessments and longitudinal follow-up are needed to validate and extend these findings.

## 5. Conclusions

The present study may suggest that higher LUS score may be associated with lower DLCO, higher DAS28-CRP, and UIP pattern in this highly selected RA cohort. In this scenario, although LUS may represent a complementary tool in the appropriate clinical context, the HRCT remains the reference method for radiological characterization in patients with RA. Further prospective and adequately powered studies in larger cohorts are warranted to support these clinical applications and to better define the role of LUS in the management of RA–associated ILD.

## Figures and Tables

**Table 1:** Baseline characteristics of study population.

Demographics	*n* = 37
Gender (M/F) *n*, %	20 (54%)/17 (45.9%)
Age, years (mean ± SD)	68.8 ± 10.4
BMI, kg/m^2^ (mean ± SD)	26.3 ± 3.9
Smoking status (former/current/never) *n*, %	20 (54%)/9 (24.3%)/8 (21.6%)
Pack years, median (IQR)	10 (22)
RA disease duration (months), median (IQR)	96 (216)
Interval DAS28-CRP—pneumological assessment (months), median (IQR)	2.9 (1.5)
Interval HRCT—LUS (months), median (IQR)	2.1 (3.0)
**Comorbidities**	
Arterial Hypertension *n*, %	16 (43.2%)
GERD *n*, %	11 (29.7%)
Obesity *n*, %	10 (27%)
Dyslipidemia *n*, %	8 (21.6%)
Diabetes *n*, %	7 (18.9%)
Atrial Fibrillation, *n* %	5 (13.2%)
Heart Failure, *n* %	2 (5.4%)
**Chest HRCT findings**	
ILD (yes/no) *n*, %	24 (64.8%)/13 (35.1%)
UIP pattern (yes/no) *n*, %	17 (45.9%)/20 (54.1%)
NSIP pattern (yes/no), *n*, %	5 (13.5%)/32 (86.5)
uILD pattern (yes/no), *n*, %	1 (2.7%)/36 (97.3%)
OP pattern (yes/no), *n* %	1 (2.7%)/36 (97.3%)
**Pulmonary function tests**	
FVC%pred (mean ± SD)	84.6 ± 18
FVC L, (mean ± SD)	2.56 ± 0.87
DLCO%pred, (mean ± SD)	61 ± 20.4
**Treatments**	
Glucocorticoids, *n* %	15 (40.5%)
**csDMARDs**	
Methotrexate, *n* %	32 (86.5%)
Hydroxycloroquine, *n* %	9 (24.3%)
Leflunomide, *n* %	7 (18.9%)
**bDMARDs**	
Abatacept, *n* %	9 (24.3%)
Etarnecept, *n* %	7 (18.9%)
Adalimumab, *n* %	5 (13.5%)
**tsDMARDs**	
Filgotinib, *n* %	5 (13.5%)
Upadacitinib, *n* %	3 (8.1%)
**Other**	
Rituximab, *n* %	7 (18.9%)
Tocilizumab, *n* %	5 (13.5%)
**Clinical Variables**	
Anti-cyclic citrullinated peptide antibodies (yes/no) *n*, %	30 (81.1%)/7 (18.9%)
Rheumatoid Factor (yes/no) *n*, %	29 (78.4%)/8 (21.6%)
DAS28-CRP median (IQR)	2.20 (1.2)
LUS score median (IQR)	22 (24)

Abbreviations: IQR, interquartile range; BMI, body mass index; GERD, gastroesophageal reflux; HRCT, high-resolution computed tomography; ILD, interstitial lung disease; UIP, usual interstitial pneumonia; NSIP, non-specific interstitial pneumonia; uILD, unclassifiable interstitial lung disease; OP, organizing pneumonia; FVC%pred, forced vital capacity percent-predicted; FVC L, forced vital capacity liters; DLCO%pred, diffusing lung capacity for carbon monoxide percent-predicted; DAS28-CRP, disease activity score28-C reactive protein; LUS score, lung ultrasound score; csDMARDs, conventional disease-modifying antirheumatic drugs; bDMARDs, biologic disease-modifying antirheumatic drugs; tsDMARDs, targeted-synthetic disease-modifying antirheumatic drugs.

**Table 2:** Uni- and multivariable logistic regression analyses assessing the association between LUS score and the UIP pattern on chest HRCT.

Variables	Univariable (OR) 95% CI	*p*-Value	Multivariable (OR) 95% CI	*p*-Value
LUS score (ref. continuous)	1.09 (1.02–1.16)	<0.01	1.07 (1.01–1.15)	0.02
**Covariates**				
Age (ref. continuous)	1.07 (0.98–1.18)	0.10	1.02 (0.92–1.14)	0.60

Abbreviations: LUS, lung ultrasound score; OR, odds ratio.

**Table 3:** Univariable and multivariable logistic regression analyses assessing the association of DAS28-CRP and the UIP pattern on chest HRCT.

Variables	Univariable (OR) 95% CI	*p*-Value	Multivariable (OR) 95% CI	*p*-Value
DAS28-CRP (ref. continuous)	3.05 (1.30–7.14)	0.01	2.35 (1.19–6.86)	0.02
**Covariates**				
Age (ref. continuous)	1.07 (0.98–1.18)	0.10	1.05 (0.96–1.12)	0.27

Abbreviations: DAS28-CRP, disease activity score 28–C reactive protein; OR, odds ratio.

## Data Availability

The data presented in this study are available on request from the corresponding author due to privacy and ethical restrictions.

## Supplementary Materials

The following supporting information can be downloaded at: https://www.mdpi.com/article/10.3390/biomedicines14102281/s1, S1: Distributions of LUS score across HRCT subgroups; S2: Association between UIP pattern and categorized LUS score and DAS28-CRP values; S3: Exploratory analysis of dichotomized LUS and DAS28-CRP scores in relation to UIP pattern; Table S1A: Exploratory analysis assessing the association between dichotomized LUS score and UIP pattern; Table S1B: Exploratory analysis assessing the association between dichotomized DAS28-CRP and UIP pattern.

## Abbreviations

ACR/CHEST	American College of Rheumatology/American College of Chest Physicians
ACR/EULAR	American College of Rheumatology/European Alliance of Associations for Rheumatology
ATS/ERS/JRS/ALAT	American Thoracic Society/European Respiratory Society/Japanese Respiratory Society/Latin American Thoracic Association
bDMARDs	Biologic disease-modifying antirheumatic drugs
BMI	Body mass index
CI	Confidence interval
CRP	C-reactive protein
csDMARDs	Conventional synthetic disease-modifying antirheumatic drugs
CTD	Connective tissue disease
DAS28-CRP	Disease Activity Score 28-C-reactive protein
DLCO	Diffusing capacity of the lung for carbon monoxide
DLCO%pred	DLCO percent-predicted
ERS/ATS	European Respiratory Society/American Thoracic Society
ERS/EULAR	European Respiratory Society/European Alliance of Associations for Rheumatology
FVC	Forced vital capacity
FVC L	FVC in liters
FVC%pred	FVC percent-predicted
GERD	Gastroesophageal reflux disease
HRCT	High-resolution computed tomography
ILD	Interstitial lung disease
IQR	Interquartile range
LUS	Lung ultrasound
LUS score	Lung ultrasound score
MDD	Multidisciplinary discussion
MDT	Multidisciplinary team
MHz	Megahertz
non-RA-ILD	ILD not related to RA
NSIP	Non-specific interstitial pneumonia
OP	Organizing pneumonia
OR	Odds ratio
PFTs	Pulmonary function tests
RA	Rheumatoid arthritis
RA-ILD	Rheumatoid arthritis–associated interstitial lung disease
ROC	Receiver operating characteristic
SD	Standard deviation
tsDMARDs	Targeted-synthetic disease-modifying antirheumatic drugs
uILD	Unclassifiable interstitial lung disease
UIP	Usual interstitial pneumonia
χ^2^	Chi-square
