# Supplementary material for: Lung Ultrasound in Rheumatoid Arthritis–Associated Interstitial Lung Disease: Association with Radiological Patterns, Lung Function Tests, and Disease Activity—A Single-Center Retrospective Cross-Sectional Study
Source: Biomedicines. 2026 Oct 8;14(10):2281. doi: 10.3390/biomedicines14102281 (PMC13701566; doi:10.3390/biomedicines14102281)
Supplement: Supplementary file 1 [file biomedicines-14-02281-s001.zip › biomedicines-4568877-supplementary.pdf]

Supplementary Material

S1. **Distributions of LUS score across HRCT subgroups.** Distribution of LUS scores according to HRCT patterns. UIP (n = 17) and NSIP (n = 5) were the most frequently observed patterns, whereas OP and uILD were each identified in one patient (n = 1). Data are presented as median (IQR) for UIP and NSIP and as median only for OP and uILD.

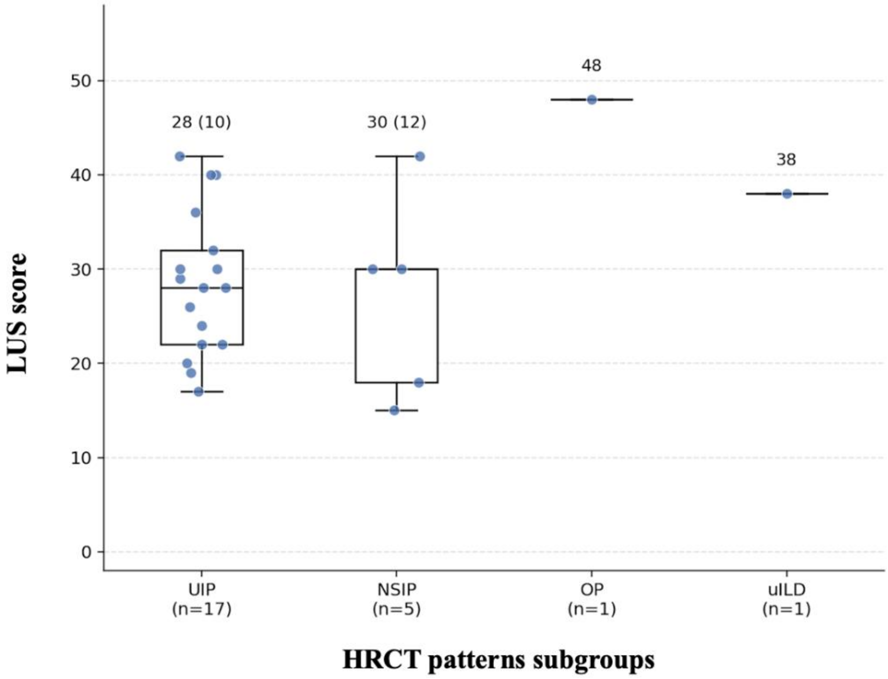


S2. **Association between UIP pattern and categorized LUS score and DAS28-CRP values.** Higher LUS score (≥ 22) and DAS28-CRP (≥ 2.2) categories were significantly associated with the presence of a UIP pattern on chest HRCT ($\chi^2 = 10.1$, $p < 0.01$; $\chi^2 = 5.45$, $p = 0.02$, respectively).

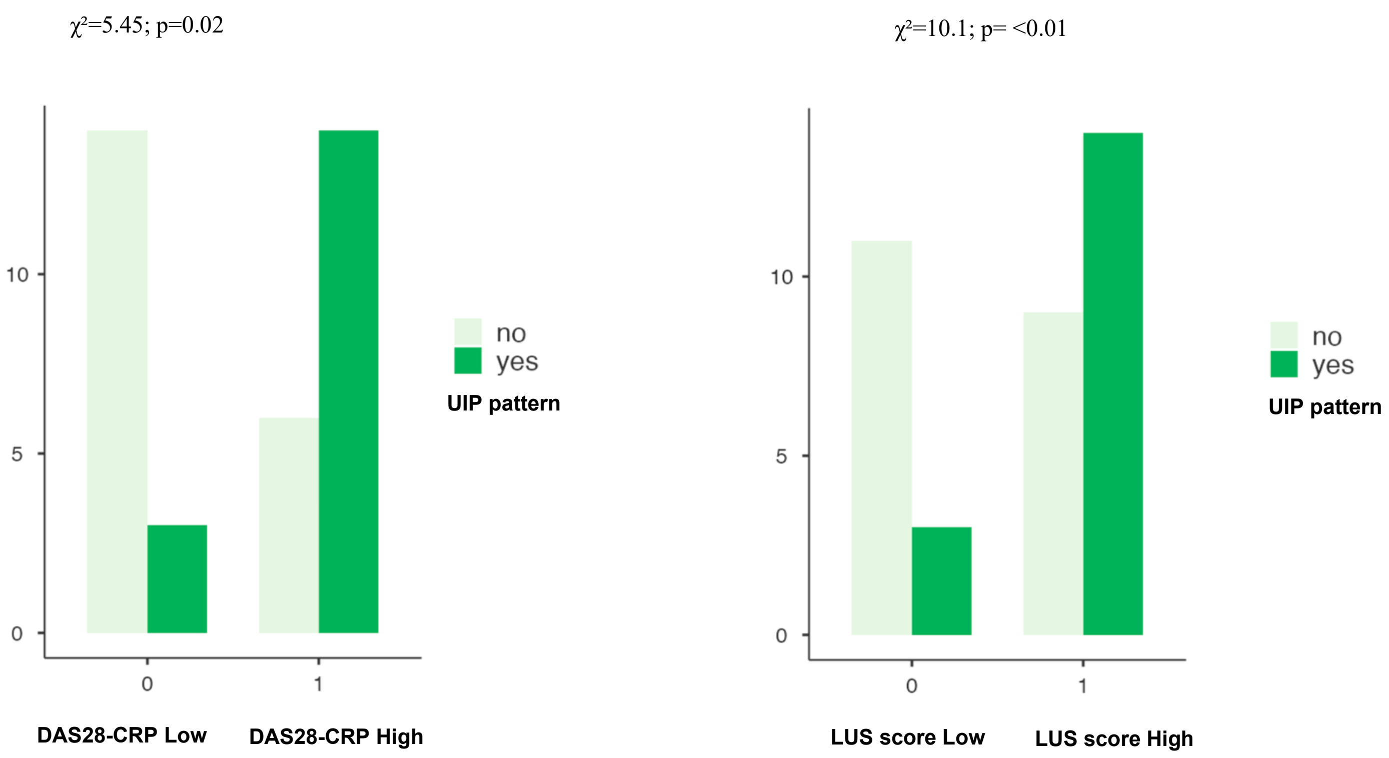


S3. **Exploratory analysis of dichotomized LUS and DAS28-CRP scores in relation to UIP pattern**. In an exploratory analysis, LUS score and DAS28-CRP were dichotomized according to their cohort-derived median values (LUS ≥22 and DAS28-CRP ≥2.20). A LUS score ≥22 was significantly associated with a UIP pattern on chest HRCT at both univariable (OR 10.89; 95% CI: 2.26–52.4; p<0.01) and multivariable analysis adjusted for age (OR 9.31; 95% CI: 1.64–52.8; p=0.01) (Table 4A). Similarly, DAS28-CRP ≥2.20 was significantly associated with UIP pattern at univariable (OR 5.70; 95% CI: 1.24–26.2; p=0.02) and multivariable analysis adjusted for age (OR 4.96; 95% CI: 1.03–23.8; p=0.04) (Table 4B).

**Table S1A.** Exploratory analysis assessing the association between dichotomized LUS score and UIP pattern.

| Variable | Univariable (OR) 95% IC | P-value | Multivariable (OR) 95% IC | P-value |
|---|---|---|---|---|
| **LUS score ≥ 22 (ref. <22)** | 10.89 (2.26 – 52.4) | <0.01 | 9.31 (1.64 – 52.8) | 0.01 |
| **Covariates** | **Univariable (OR) 95% IC** | **P-value** | **Multivariable (OR) 95% IC** | **P-value** |
| **Age (ref. continuous)** | 1.07 (0.98 – 1.18) | 0.10 | 1.01 (0.92 – 1.12) | 0.70 |

Abbreviations: LUS, lung ultrasound score; OR, odds ratio

**Table S1B.** Exploratory analysis assessing the association between dichotomized DAS28-CRP and UIP pattern.

| Variable | Univariable (OR) 95% IC | P-value | Multivariable (OR) 95% IC | P-value |
|---|---|---|---|---|
| **DAS28-CRP ≥ 2.20 (ref. < 2.20)** | 5.70 (1.24 – 26.2) | 0.02 | 4.96 (1.03–23.8) | 0.04 |
| **Covariates** | **Univariable (OR) 95% IC** | **P-value** | **Multivariable (OR) 95% IC** | **P-value** |
| **Age (ref. continuous)** | 1.07 (0.98 – 1.18) | 0.10 | 1.05 (0.97 – 1.14) | 0.18 |

Abbreviations: DAS28-CRP, disease activity score28-C-reactive protein; OR, odds ratio
